# A ’metabolic bundle‘ including Oxandrolone in optimising the metabolic status of severely burn injured patients: a retrospective analysis of the first 50 patients

**DOI:** 10.3205/iprs000143

**Published:** 2019-11-15

**Authors:** Hischam Taha, Björn Steinke, Hagen Fischer, Michael J. Malcharek, Thomas Kremer, Jochen Gille

**Affiliations:** 1 Department of Plastic and Hand Surgery with burn care unit, St. Georg Hospital gGmbH Leipzig, Germany; 2 Department of Anaesthesiology, Intensive Care Medicine and Pain Therapy, St. Georg Hospital gGmbH Leipzig, Germany

**Keywords:** burn injury, metabolic response, oxandrolone, propanolol, trace elements

## Abstract

**Objective:** Severe burn injuries are associated with a rapid escalating hypermetabolic state and catabolism of muscle mass. To ameliorate this process a standardized approach using pharmacological and non-pharmacological interventions was implemented within a single burns center. Whilst individual components of this standardized package are well documented in the literature, their collective or bundled effect has not as yet been assessed. The aim of this study was to evaluate the efficacy of this standardized bundle of metabolic modulators and assess the safety of including the anabolic steroid oxandrolone within it.

**Methods:** This retrospective observational study constituted all patients in whom the metabolic bundle including oxandrolone therapy was applied. The other elements of the metabolic bundle consisted of early surgical burn excision within seven days to completion, early active mobilization, increased ambient room temperature, expediated carbohydrate and protein rich enteral feeding with glutamine and trace element supplements (such as copper and zinc). Finally, administration of propranolol as a non-selective beta-blocker.

Data collection was through review of the patient data management system focusing on the outcome criteria and hepatic blood values.

**Results:** The study looked at fifty consecutive patients meeting the inclusion criteria. Median patient age and burned total body surface area (TBSA) were 62 years [51.75; 73] and 33.75% [24.75; 51] respectively with an abbreviated burn severity index (ABSI) of 10 [9; 10.25]. Definitive surgical burn wound excision was completed in 44 patients [88%] within 7 days. 39 patients (78%) received propranolol over a therapeutic period of 29 days [19; 44].

Glutamine was supplemented in 45 patients (90%), while zinc and copper were applied to 42 (84%) and 31 (62%) respectively. Significant low zinc values were noted at therapeutic onset (6.5 mmol/l [4.7; 7.9]) requiring sustained substitution over 37.5 days [22; 46.75]).

In respect of the inclusion criteria, all patients received oxandrolone at 20 mg/day [20; 20]. This was commenced on day 6.5 [4; 14] post burn injury and continued over 26 days [19; 31]. Despite a transitory elevation of hepatic enzyme values (ALT, GGT), these were only clinically relevant (>10 µmol/l*S) in 2.4% and 4.6% of all measurements respectively. None were sufficiently of concern to merit cessation of treatment.

**Conclusion:** The application of a standardised bundle of metabolic treatment options of severe burns injured patients is reliable, repeatable and safe. Potential concerns of oxandrolone treatment regarding hepatic compromise remain unfounded.

## Zusammenfassung

**Ziel:** Schwere Brandverletzungen sind mit der Entwicklung eines hyperdynamen Hypermetabolismus und Muskelkatabolismus assoziiert. In unserer Klinik wurde ein Maßnahmenpaket aus pharmakologischen und nichtpharmakologischen Interventionen zur Beeinflussung dieser metabolischen Veränderungen implementiert. Während für die einzelnen Interventionen zumeist aussagekräftige Studien existieren, gibt es bislang keine Untersuchung über die gebündelte Anwendung innerhalb eines Maßnahmenpakets. Ziel dieser Studie war es, die Praktikabilität und Sicherheit der Anwendung dieses Maßnahmenpakets (‚metabolisches Bündel’) unter besonderer Berücksichtigung des Anabolikums Oxandrolon zu untersuchen.

**Methoden:** In die retrospektive Observationsstudie wurden alle Patienten eingeschlossen, bei denen das metabolische Bündel einschließlich der Therapie mit Oxandrolone angewendet wurde. Das metabolische Bündel beinhaltete zusätzlich: die Nekrektomie der Verbrennungswunden innerhalb der ersten 7 Tage, die Frühmobilisation, die Erhöhung der Umgebungstemperatur, die frühe kohlenhydrat- und eiweißreiche enterale Ernährung unter Zusatz von Glutamine, die nicht-selektive Betablockade mit Propanolol sowie die Gabe von Spurenelementen (Kupfer, Zink). Die Datenerfassung erfolgte mittels automatisierter Abfragen aus dem Patendatenmanagementsystem unter Einbeziehung der Outcomeparameter sowie des Verlaufes der Leberwerte.

**Ergebnisse:** Insgesamt wurden 50 Patienten eingeschlossen. Das mediane Patientenalter und die verbrannte Körperoberfläche (VKOF) betrugen 62 Jahre [51.75; 73] bzw. 33.75% [24.75; 51] bei einem ABSI von 10 [9; 10.25]. Die definitive Nekrektomie erfolgte bei 44 Patienten (88%) innerhalb von 7 Tagen. Propanolol erhielten 39 Patienten (78%) mit einer Therapiedauer von 29 Tagen [19; 44]. Bei 45 Patienten (90%) wurde neben einer enteralen Ernährung zusätzlich Glutamin appliziert. Zink und Kupfer wurden bei 42 (84%) bzw. 31 Patienten (62%) substituiert. Dabei zeigten sich deutlich erniedrigte Zinkwerte zu Therapiebeginn (6.5 mmol/l [4.7; 7.9]) und ein anhaltender Substitutionsbedarf im Verlauf (Therapiedauer 37.5 Tage [22; 46.75]). Gemäß der Einschlusskritierien erhielten alle Patienten Oxandrolon (n=50) mit Therapiebeginn im Median an Tag 6,5 [4; 14] nach Verbrennungstrauma und einer Therapiedauer von 26 Tagen [19; 31]. Die Dosis betrug 20 mg/Tag [20; 20]. Trotz eines transitorischen Anstiegs der Leberenzyme (ALT, GGT) wurden klinisch relevante Erhöhungen (>10 µmol/l*S) lediglich in 2,4% bzw. 4.6% der Messungen beobachtet. Bei keinem Patienten waren die Erhöhungen mit der Notwendigkeit des Abbruchs der Therapie verbunden.

**Schlussfolgerung:** Die Anwendung eines standardisierten Bündels von Maßnahmen zur Beeinflussung des Metabolismus bei Schwerbrandverletzten ist praktikabel und sicher. Potentielle Bedenken gegen die Anwendung des Anabolikums Oxandrolon hinsichtlich hepatischer Komplikationen erscheinen unbegründet.

## Introduction

Large burn injuries are marked by an intensive inflammatory response, escalating to a hypermetabolic and catabolic state. The resultant progressive loss of mean body mass, if unencumbered, is associated with increased morbidity and mortality [1], [2].

The degree and duration of burn injury induced hypermetabolism far exceeds that which would be expected in comparison to that manifested by other causes of sepsis and trauma [2]. The metabolic rate can remain significantly elevated for over a year following initial injury [1], [3]. Indeed, onset of base rate metabolic increases may already be observed at 20% burned total body surface area (TBSA) [4].

The most important hormonal mediated stress response is delivered through cortisol and catecholamine [1], [4], [5], [6]. These are supplemented by free radicals, arachidonic acid and mediators of inflammation such as cytokines, tumor necrosis factor alpha (TNFα) and interleukins 1 and 6 (IL1, IL6) [7], [8]. At the cellular level oxygen demand is increased through ATP-consumption and heat generation [1], [2]. It is this greater understanding of the metabolic pathways involved that has permitted an emphasis shift from mortality to one of morbidity reduction through amelioration of the hypermetabolic state [1], [2].

The therapeutic modulation of metabolic demand, in particular the reduction of catabolic processes, applies pharmacological and non-pharmacological concepts. The later includes definitive and decisive surgical management of the burn injury [9], increase in ambient room temperature (31.5±0.7°C), early high calorific and protein rich enteral feeding and intensive physiotherapy. Pharmacological therapeutic applications include insulin, propranolol and oxandrolone administration [1], [2], [9].

The efficacy and safety of oxandrolone is now well established in the literature [10] and specifically strongly recommended as an essential component of the European Society for Clinical Nutrition and Metabolism (ESPEN) 2013 guidelines on nutritional therapy in burns [11]. Oxandrolone has been shown to improve lean muscle mass and net protein balance during the catabolic phase after severe burns and accelerates wound healing shortening lengths of hospital admission [5], [12], [13]. Despite the widespread acceptance in the literature of the use of oxandrolone as part of a severe burns treatment regime, its widespread use continues to meet reservation, as is the case in Germany. This arises through concerns of hepatotoxicity and a perceived off-licence use within the Federal Republic [14].

A multifaceted approach, collating the current evidence based best therapeutic practices in the treatment of burns injured patients has been applied as a standard operating procedure in the form of a therapeutic bundle. The current paper presents the outcome of the first fifty patients to whom this concept of a ’metabolic bundle‘ including the use of oxandrolone has been applied.

## Methods

### Subjects and study design

The current single center study was retrospective and observational, based on 50 consecutive patients admitted to a regional burns center in Germany over an eight-year period to whom a standardized metabolic bundle treatment plan including oxandrolone was applied. Pediatric patients (<18 years of age) were excluded from the study. 

### Ethical approval

Ethical approval was obtained from the local German Federal State Chamber of Medicine, on 30^th^ September 2015 (EK-BR-70/15-1/291514).

### Burn care protocol

Patient admission to the burns center, triggers the appropriate standardized burn care protocol and, if necessary, the initial treatment of shock. 

Fluid resuscitation commenced via Parkland’s formula (4 ml/kg BW/% TBSA) was subsequently titrated to maintain a urinary output of 0.5 ml/kg BW/h. Cardiovascular support, where indicated, was provided by noradrenalin and 20% human albumin infusion to maintain a concentration of 25.0 g/l.

Surgical burn wound management through excision and skin grafting was initiated within 72 hours of burn injury. Burn wound excision was limited to 20% TBSA at a single operating session, minimizing the traumatic insult. Larger burns thus undergo staged surgical procedures at 2–3 day intervals allowing physiological stabilization until complete burn wound excision and definitive wound closure was achieved. 

### Oxandrolone and the metabolic bundle

Acute severe burn injury admissions ≥30% burned TBSA and/or abbreviated burn severity index (ABSI) ≥8 trigger the initiation of a multidisciplinary approach involving intensive care, surgical and allied healthcare professional input incorporated as a ‘metabolic bundle’. This may be broken down into pharmacological and non-pharmacological components (Table 1 (Tab. 1)).

Early specialist and supplemented enteral nutrition commenced ideally within six hours of admission in the absence of contraindications as appropriate. Upon adequate oral intake, protein was supplemented through milk powder (84 g Protein/100 g). In cases of inadequate oral intake, nasogastric feeding of specialized high protein and carbohydrate with a reduced fat content was used (protein 6.7 g/100 g, carbohydrate 17.7 g/100 g and fat 3.7 g/100 g). This corresponds to 20.5%, 54.1% and 25.4% of the energy intake respectively. Energy requirement was calculated daily as 25–30 kcal/kg body weight.

### Data collection

Data was retrospectively collected from patient medical records and electronic patient data management systems (ICU Data^®^, IMESO, Gießen, Germany). Demographics, etiology of burn injury, injury characteristics, calculation of Baux score and abbreviated burn severity index (ABSI), packed red blood cell and fresh frozen plasma transfusion requirements, number of operations to definitive burn wound treatment and length of hospital admission were recorded.

Additional data representing the clinical course, including complications, were registered (pneumonia, requirement and total duration of ventilation, acute renal failure requiring continuous renal replacement therapy, sepsis and mortality). To monitor potential detrimental side effects of oxandrolone treatment, regular blood analysis of liver enzymes such as alanine transaminase (ALT, upper limit of normal serum concentration <0.85 µmol/l*s) and gamma glutamyl transferase (GGT, upper limit of normal serum concentration <1.02 µmol/l*s) were recorded. Trace element supplementation was evaluated through documentation of zinc (normal limits 9–18 mmol/l) and copper (normal limits 11–22 µmol/l) concentrations. Timing of onset, frequency and duration of therapy options, surgical, pharmacological (propranolol and oxandrolone) were also recorded.

### Statistical analysis

A descriptive presentation of the results through interquartile ranges and medians, interquartile range 25%–75% [IQR] and 50^th^ percentile respectively, were employed graphically in scatterplot and box diagrams. Collated data underwent statistical analysis and graphic presentation using a commercial software package (SPSS^®^ Version 24, SPSS Inc. Chicago, IL, USA). Non-parametric Wilcoxon tests were performed for comparisons between two time points. The alpha level of the study was P=0.05.

## Results

### Patients and injury characteristics

The study included 50 patients. Demographic data and injury characteristics are summarized in Table 2 (Tab. 2).

### Timing of surgery, clinical complications and outcomes

Complete surgical burn excision within the first 7 days post injury was achieved in 44 patients (88%). Of these, complete surgical excision was achieved at a single operation in 21 (42%) patients, 24 (48%) in two operations, whilst 5 (10%) patients required three procedures to complete definitive burn debridement. Salient outcome parameters are listed in Table 3 (Tab. 3).

### Oxandrolone and pharmacological interventions

Oxandrolone therapy commenced on day 6.5 [4; 14] for a duration of 26 days [19; 31]. Glutamine was administered to 45 (90%) patients, whilst trace element substitution of zinc and copper was provided to 42 (84%) and 31 (62%) patients respectively and propranolol to 39 (78%). The onset, duration and dosage of treatment aspects are summarized in Table 4 (Tab. 4).

### Zinc and copper levels

**Figure 1:**
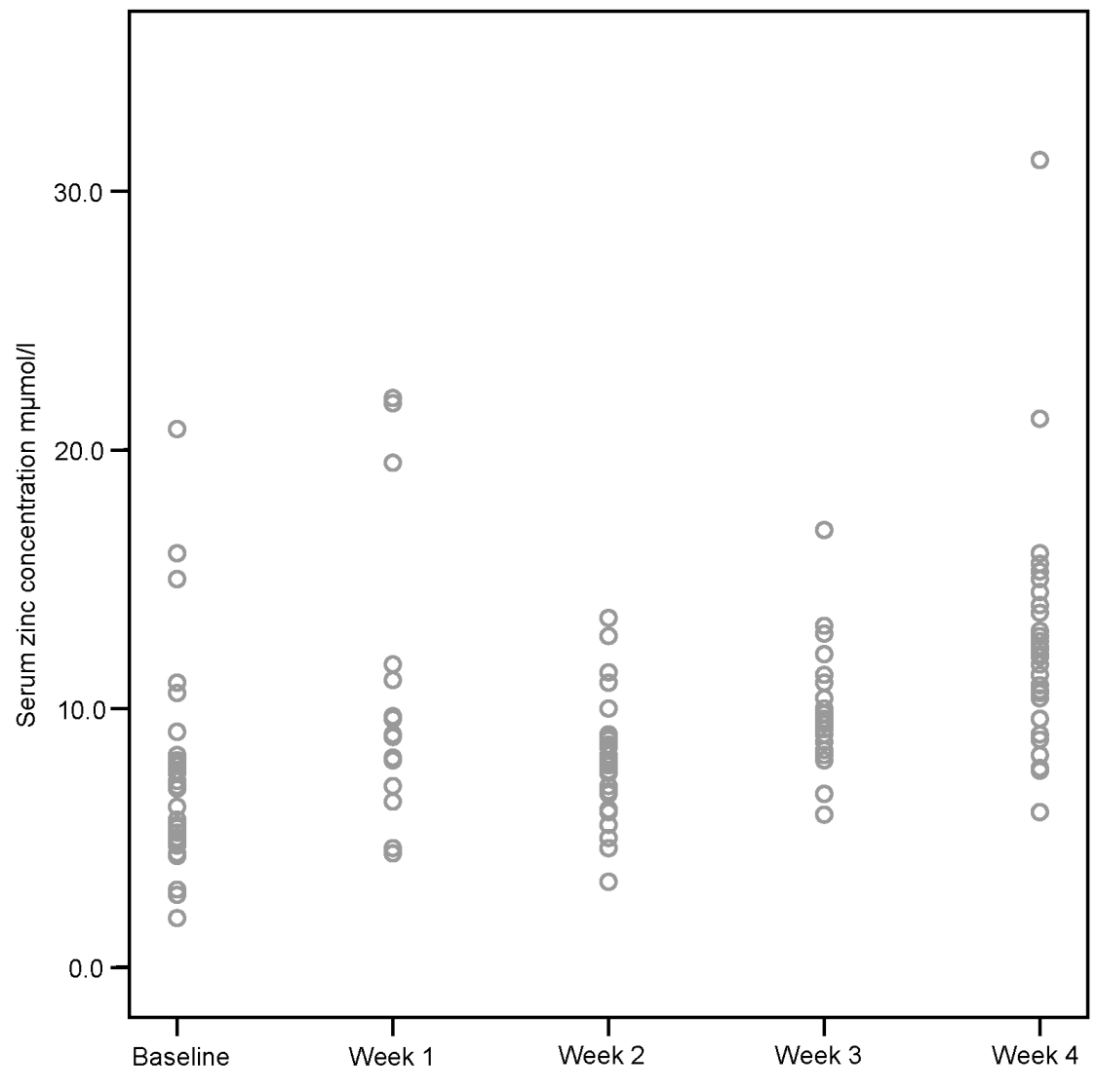
Scatterplot of zinc concentrations during four weeks after admission (normal limits 9–18 mmol/l). [T-Test, 2-sided, significance level a<0.05; P-values (b=baseline, w=week) b:w1=0.018, b:w3=0.004, b:w4<0.001, w2:w3<0.001, w2:w4<0.001, w3:w4=0.004]

Zinc levels monitored within the first three days of admission were recorded as 6.5 mmol/l [4.7; 7.9]. The weekly concentration levels changed as follows during the admission period. Week 1 8.5 mmol/l [7; 11.25], Week 2 7.9 mmol/l [7; 9], Week 3 9.7 mmol/l [8.8; 11.2], Week 4 12 mmol/l [10.4; 14]. A statistically significant (P<0.05) measured increase between the weeks was noted with the exception of the week 2 to 3 interphase (Figure 1 (Fig. 1)).

**Figure 2:**
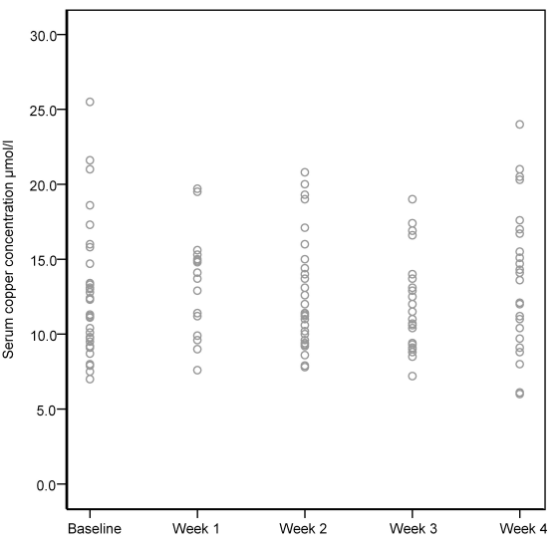
Scatterplot of copper concentrations during four weeks after admission (normal limits 11–22 µmol/l). [Wilcoxon-Test, 2-sided, significance level α<0.05, p-value: no differences between baseline and weeks and between weeks p>0.05]

Copper blood concentration levels monitored at admission were 11.1 µmol/ l [8.7; 13.4], in Week 1 13.3 µmol/l [10.9; 15.1], in Week 2 11.3 µmol/l [10; 15], in Week 3 11 µmol/l [9.3; 13.7] and in Week 4 12.10 µmol/l [9.7; 16.7]. There was no statistically significant change in concentration levels between the weeks (Figure 2 (Fig. 2)).

### Levels of ALT and GGT

ALT and GGT levels were measured 170 and 174 times respectively during a four treatment week period. 70.6% (120) of ALT values and 22.4% (39) of GGT values were within normal parameters.

**Figure 3:**
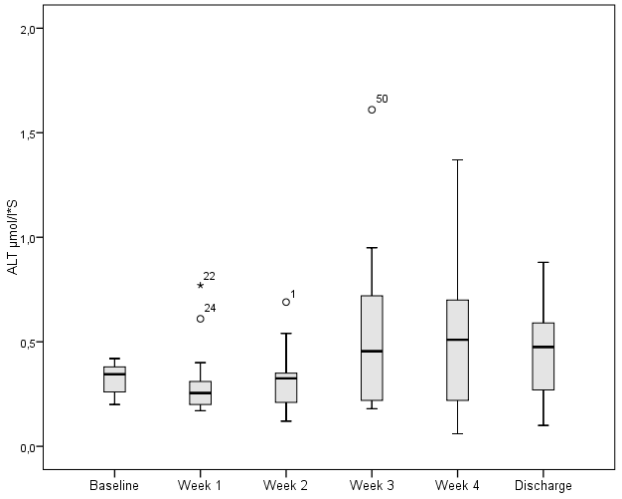
Serum alanine transaminase (ALT) concentration during period treated with oxandrolone (upper limit of normal serum concentration <0.85 µmol/l*S). [T-Test, 2-sided, significance level α<0.05, P-values (b=baseline, w=week) b:w1=0.132, b:w2=0.264, b:w3=0.113, b:w4=0.004, b:discharge=0.027; w1:w2=0.314, w1:w3=0.162, w1:w4=0.100, w1:discharge=0.420, w2:w3=0.978, w2:w4=0.438, w2:discharge=0.364, w3:w4=0.228, w3:discharge=0.198, w4:discharge=0.084]

**Figure 4:**
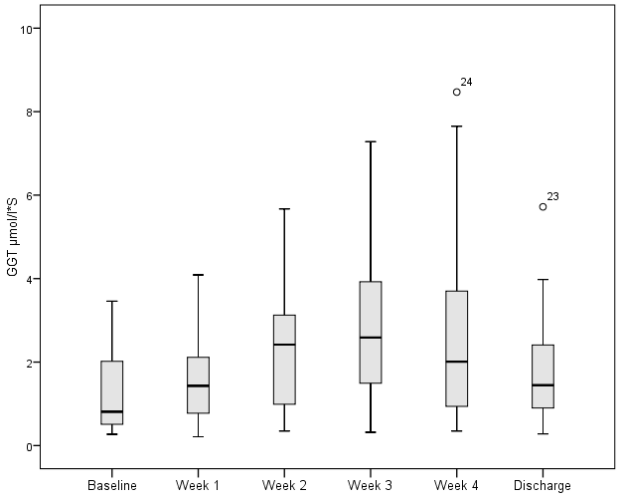
Serum gamma glutamyl transferase (GGT) concentration during period treated with oxandrolone (upper limit of normal serum concentration <1.02 µmol/L*S). [T-Test, 2-sided, significance level α<0.05, P-value (b=baseline, w=week) b:w1= 0.063, b:w2=0.000, b:w3=0.002, b:w4=0.024, b:discharge=0.079, w1:w2=0.001, w1:w3=0.002, w1:w4=0.048, w1:discharge=0.262, w2:w3=0.201, w2:w4=0.955, w2:discharge=0.461, w3:w4=0.087, w3:discharge=0.253, w4:discharge=0.680]

The respective distribution for ALT and GGT over the first four weeks were: 89.4% (n=152) and 44.8% (n=78) <2 µmol/l*S; 8.2% (n=14) and 50.6% (n=88) 2–10 µmol/l*S and 2.4% (n=4) and 4.6% (n=8) >10 µmol/l*S. Figure 3 (Fig. 3) and Figure 4 (Fig. 4) illustrate the weekly values of ALT and GGT during oxandrolone therapy including admission and discharge levels. 

The degree of increase was classified according to the defined range values. ALT and GGT were respectively distributed as follows: 89.4% (n=152) and 44.8% (n=78) <2 µmol/l*S, 8.2% (n=14) and 50.6 % (n=88) 2–10 µmol/l*S, 2.4% (n=4) and 4.6% (n=8) >10 µmol/l*S. No patients demonstrated sufficient elevated liver values to a degree that required cessation of oxandrolone therapy.

## Discussion

The current study, is the first in the literature to the authors knowledge, to explore the application of a standardized bundle of metabolic treatment options of severe burns injured patients. All elements of the multifaceted ’metabolic bundle‘ were heavily applied in the therapeutic process (Table 4 (Tab. 4)) and represent current best practice.

The use of oxandrolone as part of a therapeutic bundle to modulate the metabolic response in burn injured patients. In the context of a German burns centre the use of oxandrolone may be considered as safe. Albeit that a transitory increase in hepatic enzyme (ALT, GGT) levels was noted (Figure 3 (Fig. 3) and Figure 4 (Fig. 4)), clinically relevant levels (>10 µmol/l*S) only occurred in 2.4% and 4.6% of measurements respectively. Thus, therapeutic termination remained unnecessary in the absence of critical increases of hepatic enzymes. This corresponds to the lack of hepatic dysfunction found in a burns injured group treated with oxandrolone by McCullough et al. [15] and Li’s metanalysis more recently [10]. Transitional changes in hepatic levels may therefore be considered as an indication of the injury severity and of possible complications as a consequence. Within the current cohort, these relevant increases can be rationalised through episodes of sepsis or multiorgan failure. Oxandrolone is not currently licenced for clinical use within the German Federal Republic. This has led to uncertainties amongst clinicians with regard to its application in the treatment of burns injured patients in the German context. Federal legal clarification has nevertheless been provided to facilitate its and the use of other medical pharmaceuticals not domestically available but imported into Germany [16]. The German working group for burn injury medicine, Deutsche Gesellschaft für Verbrennungsmedizin (DGV) published a document outlining their position and explicit support for the use of oxandrolone in severe burn injury patients [17]. The authors’ experience of the logistical practicalities that have led to time delays between one to four weeks, lead the authors to recommend pre-stocking a limited quantity in advance of need.

Propranolol used in approximately 80% of study patients, reduces oxygen demand and the endogenous catecholamine induced inflammatory response [4], [18]. In the current study, propranolol was only administered after initial definitive burn wound excision with the understanding that propranolol induced peripheral vasoconstriction would be detrimental to the burns wound outcome, extending the zone of stasis according to Jackson’s model [19]. This was in line with the published recommendations of ESPEN to commence at the end of the first week [11]. In contrast Ali and colleagues supported the even earlier application of propranolol within 48 hours after burn injury by demonstrating reduced intraoperative blood loss during burn excision and skin grafting and improved wound healing [20]. This would lead one to consider a clear benefit of early propranolol administration immediately after the initial burns shock phase. Furthermore, to ESPEN’s recommendation, in North America 60% of respondents of American burns centres use propranolol routinely but its application is greatly varied with respect to dosage, duration and outcome measures [21]. These highlight the need and opportunity for further studies to drive consensus guidelines.

In combined use with oxandrolone in children, Guillory and colleagues [22] looked at propranolol plasma concentrations, half-life and cardiological effect such as heart rate, concluding no influence on pharmacodynamics. A summative benefit of the two medications has not been shown to date.

Enterally administered glutamine is a significant element of the metabolic bundle in 90% of patients also treated with oxandrolone. Published literature in this regard is currently conflicting. The ESPEN guidelines strongly suggest considering glutamine supplementation [11]. In the critically ill, glutamine showed no benefit in overall mortality nor length of stay [23]. Possible increased mortality rates in non-burns critically ill patients has led to an increased reserved approach to glutamine use, in particular those with renal disease [24], [25]. Conversely, in burn injured critically ill patients a meta-analysis conferred benefits in mortality rates, reduced length of stay and lower infection mediated complications [26]. The authors do caution about the scarcity of data and recommended need for greater prospective randomised trials. The ‘RE-ENERGIZE’ study, a contemporaneous international multicentre trial in the recruitment phase, wishes to address these issues [27].

Zinc concentrations during the first three days following admission were significantly reducing leading to 84% of patients requiring zinc trace element substitution. This need remained for several weeks. Two thirds of patients received copper trace element substitution although the majority of levels remained within but at the lower boundary of normal levels. Duration of copper substitution was shorter than for zinc at 21 compared to 37.5 days. The impact of trace element loss persists via open burn wounds losing between 20–40% of copper and 10% of zinc reserves [28]. If not substituted patients are put at risk of a variety of clinical complications such as wound healing delay, immunosuppression and cardiovascular disease [2], [28], [29].

A non-pharmacological element of the metabolic bundle is the completion of early burn wound debridement and definitive wound closure within 7 days of injury. This was achieved in 88% of patients. In this manner inflammation, hypermetabolism and infection induced sepsis may have been reduced. However, the physiological impact and distinction between early burn wound excision and early wound closure may also be more closely analysed. The benefit of early burn wound excision is well documented and accepted [30], [31]. Not so clearly defined is the benefit and timing of burn wound cover definitively with autograft or as a staged intervention using cadaveric skin or dermal substitutes prior to autograft skin closure. 

The current study is limited by its retrospective nature, leaving questions unanswerable with hind sight due to insufficient or incomplete data collation such as dynamic variation in lean muscle mass and calorific intake. Nutrition, followed a calculated standard form. Energy consumption via indirect calorimetry is only available via intubated patients within the burns centre. As a consequence calorific measurements remained sporadic, and conclusions cannot be drawn. Vitamin D substitution was not considered standard practice for the full duration of the study, but following upon Rousseau’s paper [32], vitamin D has been lately included as part of the metabolic bundle. The lack of a control group to this study is an understandable limitation. Given the evidence based nature of the metabolic bundle components and their standing as best practice, a control group is prospectively already eliminated upon ethical grounds. Recognition of the extraordinarily low group mortality rates (6%) with an ABSI 10 nevertheless allows the study to be favourably brought into perspective when compared to larger contemporaneous literature [33].

In conclusion, the application of a standardised bundle of metabolic treatment options of severe burns injured patients is reliable, repeatable and safe. Potential concerns of oxandrolone treatment remain unfounded.

## Notes

### Competing interests

The authors declare that they have no competing interests.

## Figures and Tables

**Table 1:**
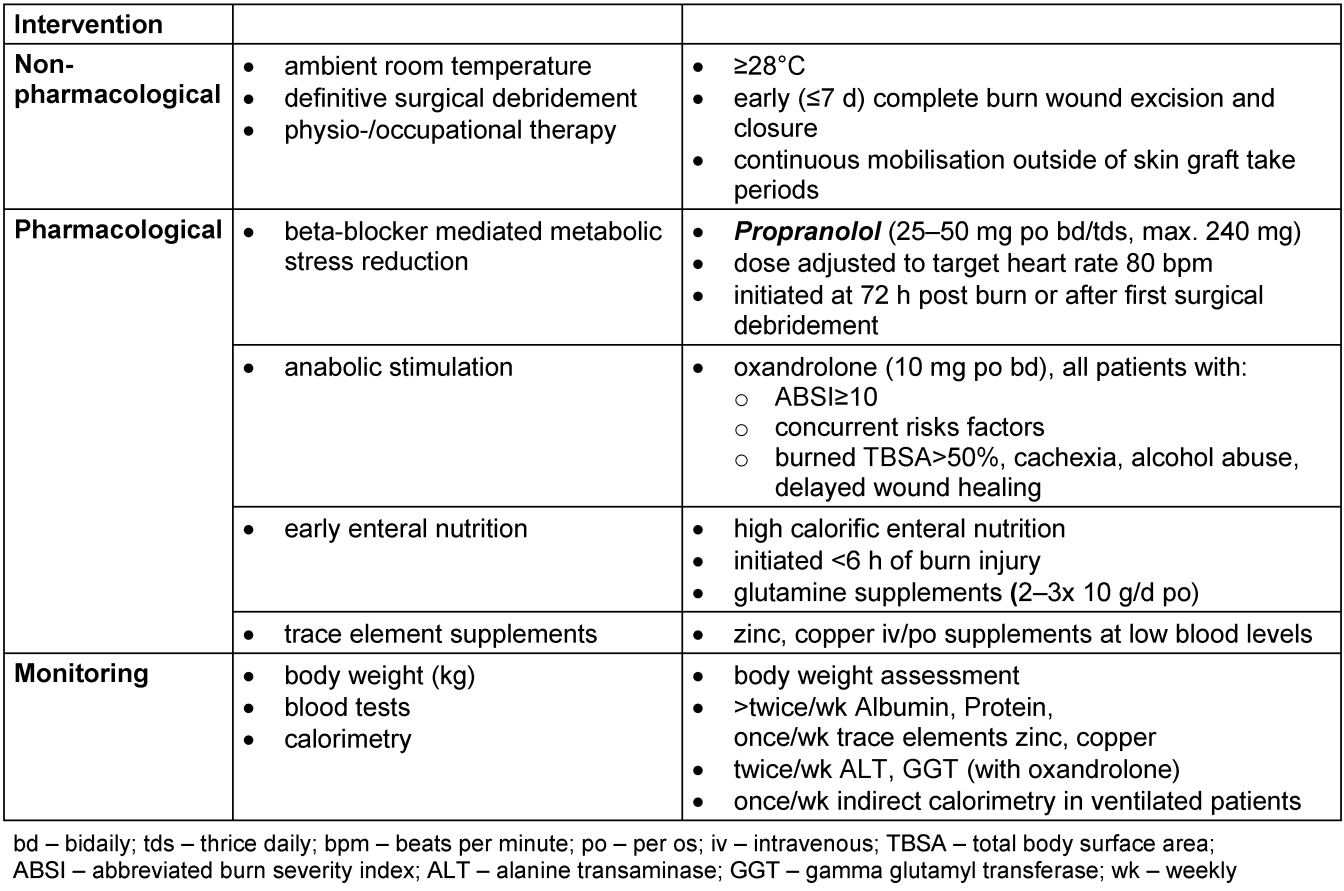
Metabolic bundle

**Table 2:**
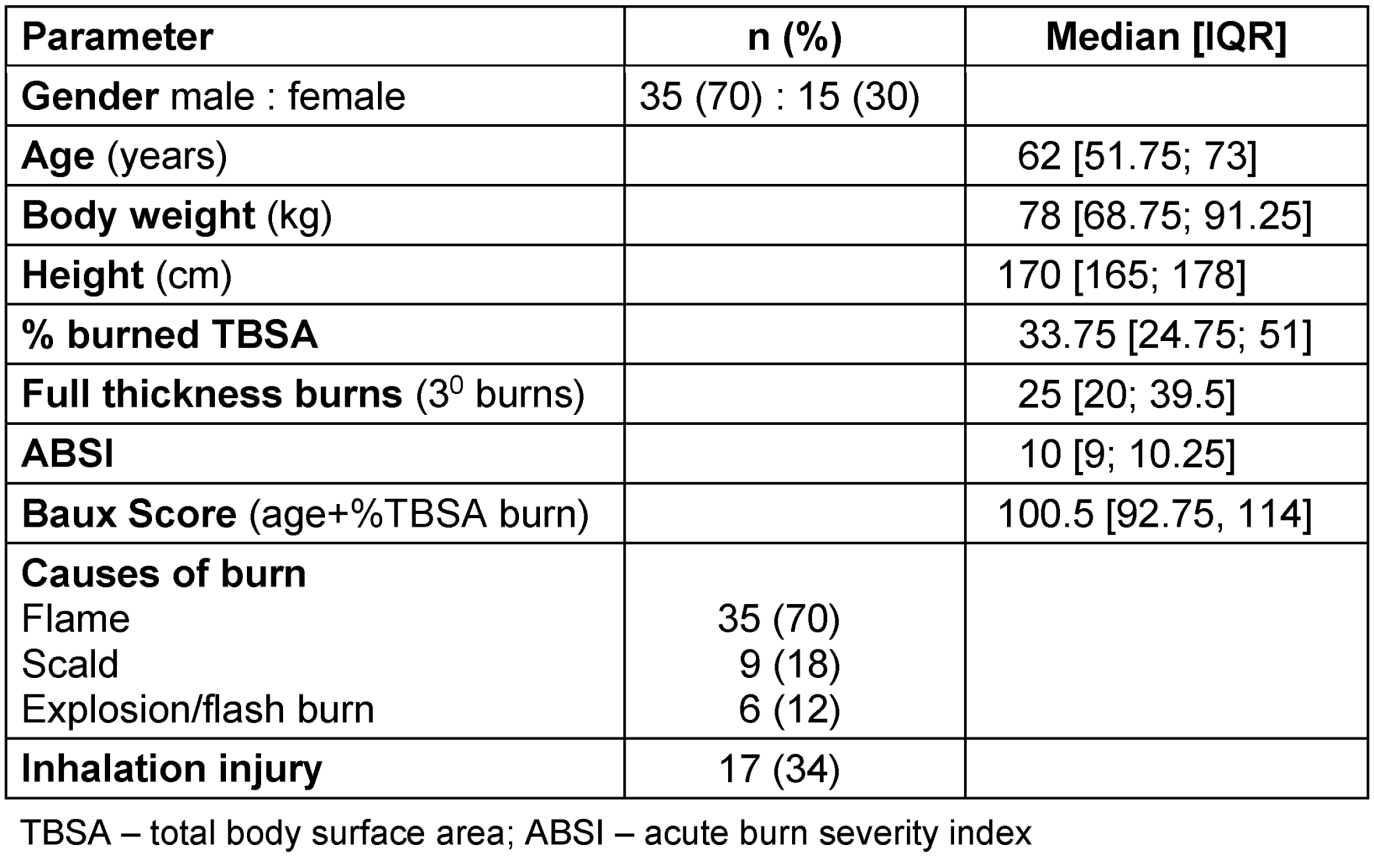
Patients (n=50) and injury characteristics

**Table 3:**
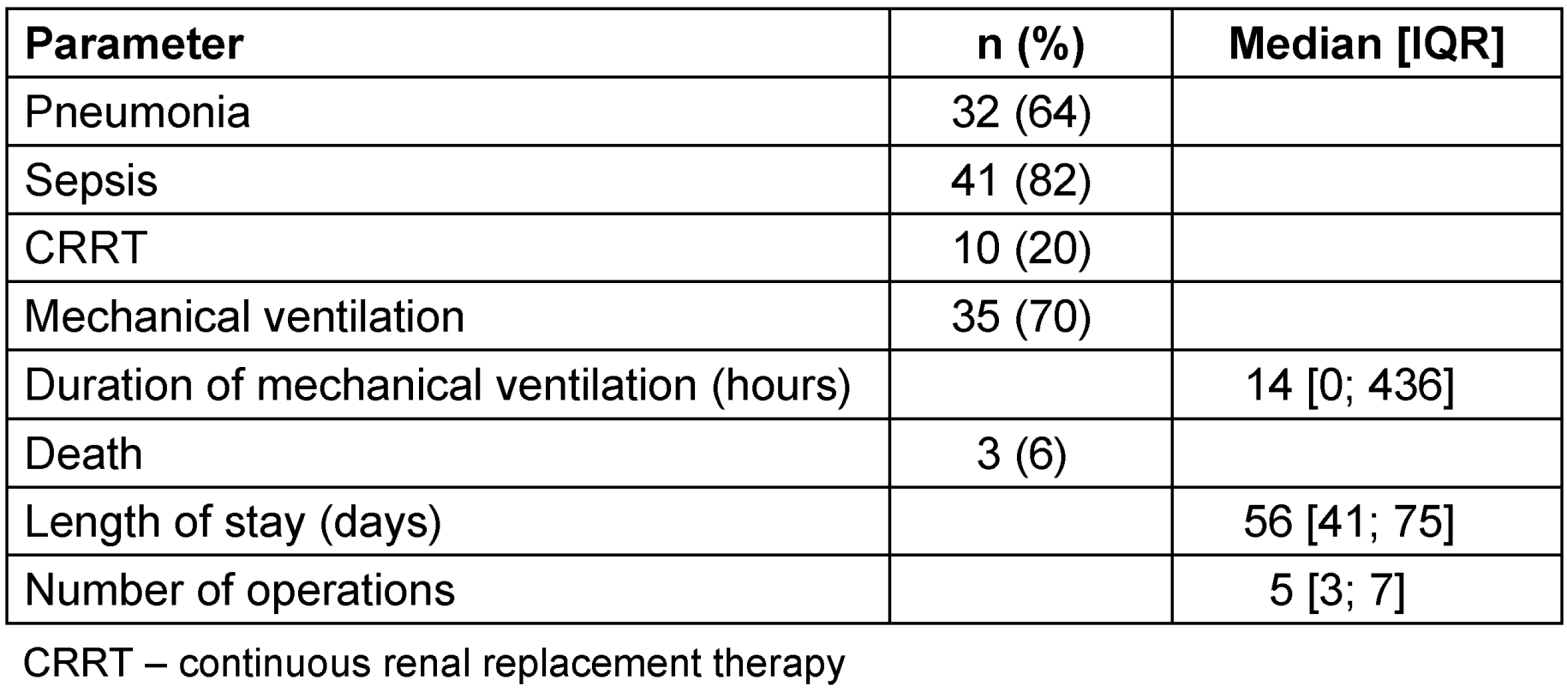
Outcome parameters of patients (n=50)

**Table 4:**
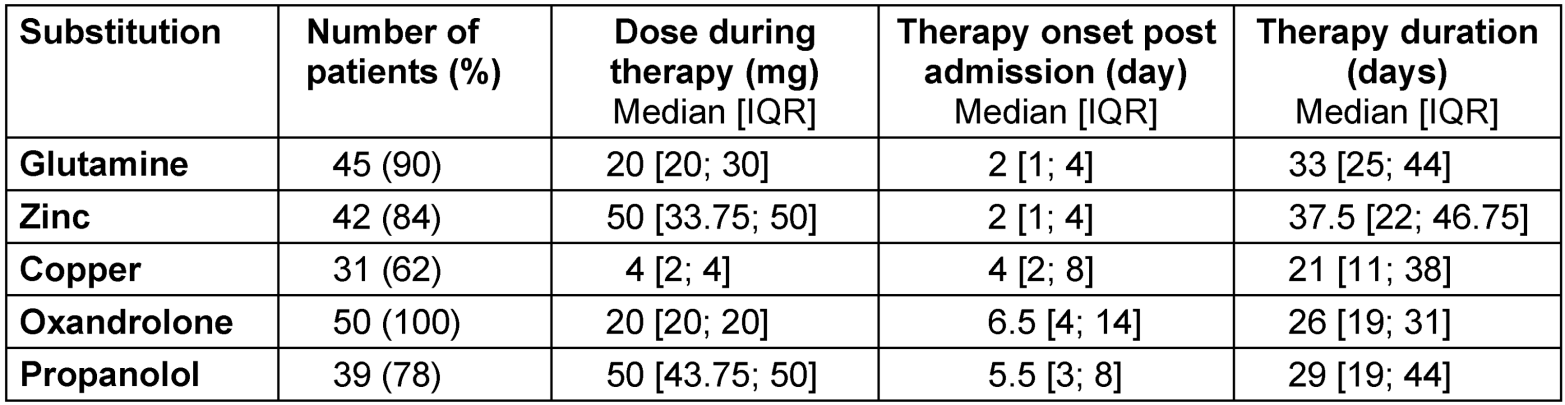
Glutamine, zinc, cooper, oxandrolone and propranolol substitution dose
